# Digital Watermarking as an Adversarial Attack on Medical Image Analysis with Deep Learning

**DOI:** 10.3390/jimaging8060155

**Published:** 2022-05-30

**Authors:** Kyriakos D. Apostolidis, George A. Papakostas

**Affiliations:** MLV Research Group, Department of Computer Science, International Hellenic University, 65404 Kavala, Greece; kyriapos1@cs.ihu.gr

**Keywords:** medical image analysis, deep learning, computer vision, adversarial attack, watermarking, robustness

## Abstract

In the past years, Deep Neural Networks (DNNs) have become popular in many disciplines such as Computer Vision (CV), and the evolution of hardware has helped researchers to develop many powerful Deep Learning (DL) models to deal with several problems. One of the most important challenges in the CV area is Medical Image Analysis. However, adversarial attacks have proven to be an important threat to vision systems by significantly reducing the performance of the models. This paper brings to light a different side of digital watermarking, as a potential black-box adversarial attack. In this context, apart from proposing a new category of adversarial attacks named watermarking attacks, we highlighted a significant problem, as the massive use of watermarks, for security reasons, seems to pose significant risks to vision systems. For this purpose, a moment-based local image watermarking method is implemented on three modalities, Magnetic Resonance Images (MRI), Computed Tomography (CT-scans), and X-ray images. The introduced methodology was tested on three state-of-the art CV models, DenseNet 201, DenseNet169, and MobileNetV2. The results revealed that the proposed attack achieved over 50% degradation of the model’s performance in terms of accuracy. Additionally, MobileNetV2 was the most vulnerable model and the modality with the biggest reduction was CT-scans.

## 1. Introduction

The evolution of deep learning and computer hardware has helped computer vision applications become reality. Some disciplines that use DL for computer vision tasks are robotics [1], image quality assessment [2], biometrics [3], face recognition [4], image classification [5], autonomous vehicles [6], etc. One of the most important applications in CV is medical image analysis, where usually DL models were trained to diagnose or predict several diseases from numerous modalities such as MRI, CT-scans, X-rays, Histopathology images, etc. Because of DL success, it has become a useful supportive tool for doctors through medical image analysis as it saves significant time from doctors’ tasks. 

Despite DL success, recent studies proved that these models can be easily fooled by imperceptibly perturbating images [7]. According to Goodfellow et al. [8], these attacks decrease the model’s efficiency due to its linearity. Adversarial attacks are divided into three main categories. The first is “white-box attack” in which attackers know the structure and the parameters of the model. The second is “grey-box attack” where attackers know only the model’s structure, and the third is “black-box attack” in which attackers know nothing about the model. Additionally, there are targeted and untargeted attacks. In the former, attackers want to misclassify the input sample in a specific class, while in the latter they just want the sample data to be misclassified. Some of the most known adversarial attacks are Fast Gradient Sign Method (FGSM) [8], Projected Gradient Descent (PGD) [9], Jacobian-based Saliency Maps Attacks (JSMA) [10], and Carlini & Wagner (C&W) [11]. Defense in adversarial attacks can be done in two ways: data level defense and algorithmic level defense. In the first category belong the adversarial training [8] and preprocessing and postprocessing methods [12], while in the second category, some methods modify the model’s architecture, classifier, and capacity [9].

This phenomenon raises questions about the safety of computer vision in medicine, as a wrong diagnosis or prediction can cost a human life. There are several attacks and defenses in medical image analysis [13] which can be exploited by the research community in order to develop models and methods that overcome this challenge. In this paper, we propose a new black-box attack, which is based on a digital watermarking methodology. When handling medical images, the main priority is to ensure that the patient’s details are protected and remain hidden from any forgery by unauthorized persons. That is why the main concern of electronic medical systems is the integration of a standard solution for maintaining the authenticity and integrity of medical images [14]. Digital watermarking is the main solution for this issue. Watermarking enters patients’ information in an invisible way, and usually this is done in binary format. This procedure is called watermark embedding. The watermark embedding must be robust because information should be extracted correctly, even if the image is attacked. 

In this paper, we bring to light that watermarking could be a serious problem because it is used for safety reasons, but we show that it can damage the performance of the decision models. In this context, we applied digital watermarking in three modalities: MRIs for brain tumor classification, X-rays for COVID-19, Pneumonia and Normal classification, and CT-scans for COVID detection in lungs. Experiments showed that the proposed watermarking attack can importantly decrease the performance of the models. The MobileNetV2 model was the most vulnerable, while DenseNets were more robust. Furthermore, the lowest values of the watermarking control parameters were able to significantly reduce the accuracy of models in CT-scans. The proposed attack reduced the accuracy by almost 50%. The rest of this paper is organized as follows. Section 2 presents related studies from the literature that applied attacks on medical images. In Section 3, a background of the applied moment-based watermarking method is provided. Section 4 provides details about implementation such as models, datasets, and parameters. Finally, Section 5 concludes this study.

## 2. Related Works

In recent years, several adversarial attacks for medical images have been proposed. Some studies have experimented with existing attacks on medical images, while others create attacks exclusively for medical images. Yılmaz et al. [15] applied FGSM attack on mammographic images. They used “Digital Database for Screening Mammography” (DDSM), which consists of normal and cancerous images. The accuracy decreased up to 30% while the Structural Similarity Index SSIM index fell below 0.2. Pal et al. [16] applied FGSM attack on X-rays and CT-Scans for COVID-19 detection. They used VGG16 and InceptionV3 models, showing that these models are vulnerable as the accuracy has decreased up to 90% in VGG-16 and up to 63% in InceptionV3. Paul et al. [17] attacked on NLST dataset using the white-box FGSM and the black-box One-pixel attacks. FGSM reduced the model’s accuracy by 36% while One-pixel by only 2–3%. Huq and Pervin [18] applied the FGSM and PGD attacks on dermoscopic images for skin cancer recognition. The model’s performance decreased by up to 75%. Some of the most known white-box attacks, FGSM, PGD, C&W, and BIM, were tested on three datasets with ResNet50. In some cases, the performance of the model decreased by 100% [19]. Ozbulak et al. [20] proposed a targeted attack for medical image segmentation, which is named Adaptive Segmentation Mask Attack (ASMA). This attack creates imperceptible samples and achieves high Intersection-over-Union (IoU) degradation. Chen et al. [21] proposed an attack for medical image segmentation by generating adversarial examples using geometrical deformations to model anatomical and intensity variations. Tian et al. [22] created an adversarial attack that is based on the phenomenon of bias field which can be caused by the wrong acquisition of a medical image, and it can affect the efficacy of a DNN. Kügler et al. [23] investigated a physical attack on skin images by drawing dots and lines with pen or acrylic on the skin. Shao et al. [24] proposed a white-box targeted segmentation attack, which is a combination of adaptive segmentation mask and feature space perturbation in order to create a Multi-Scale Attack (MSA). The authors used the gradient of the last layer and of the middle layer in order for perturbation to be small. Yao et al. [25] proposed a Hierarchical Feature Constraint (HFC) method that can be added to any attack. Adversarial attacks are detected easier in medical images than in natural images, and this method helps attacks to hide adversarial features in order for them to not be easily detected.

## 3. Materials and Methods

Image moments are one of the most important descriptors of the content of images and they have been used in several research fields such as pattern recognition [26], computer vision [27], and image processing [28]. In the past years, researchers developed orthogonal moments, which are used as kernel function polynomials with orthogonal basis. That means different moment orders describe different parts of images, which results in a minimum of information redundancy. Some well-known moment families are Zernike [29], Tchebichef [30], and Krawtchouk [31]. The watermarking method we applied used Krawtchouk moments due to its robustness under signal processing attacks.

### 3.1. Krawtchouk Moments

The Krawtchouk orthogonal moments are a family of high-resolution moments defined in the discrete domain, which was introduced into the image analysis by Yap et al. [31]. Krawtchouk moments use the discrete polynomials Krawtchouk, which have the following form,

(1) Kn(x;p,N)=F2 1(−n,−x;−N;1p)=∑k=0Nak,n,pxk

where *x*, *n* = 0,1,2, …, *N, N* > 0, *p* ∈ (0, 1) and F2 1 is the hypergeometric function.

However, using Equation (1) occurred numerical fluctuations and a more stable version of them, the weighted Krawtchouk polynomials, was used,

(2) $$\bar{K}n\left(x;p,N\right)=Kn\left(x;p,N\right)\sqrt{\frac{w\left(x;p,N\right)}{\rho \left(n;p,N\right)}}$$

where ρ(n;p,N), is the norm of the Krawtchouk polynomials,

(3) $$\rho \left(n;p,N\right)={\left(-1\right)}^{n}{\left(\frac{1-p}{p}\right)}^{n}\frac{n!}{{\left(-N\right)}_{n}},\;n=1,\;\ldots ,\;N$$

and w(x;p,N), the weight function of the Krawtchouk moments

(4) $$w\left(x;p,N\right)=\left(\begin{array}{c} N\\ x \end{array}\right)p^{x}{\left(1-p\right)}^{N-x})\;$$

In Equation (3) the symbol (.)*_n_* corresponds to the Pochhammer symbol, which for the general case is defined as ${\left(a\right)}_{k}=\left(a+1\right)\ldots \left(a+k+1\right)$.

Based on the above definitions, the orthogonal discrete Krawtchouk image moments of (*n* + *m*)*^th^* order, of an NxM image with intensity function *f*(*x*, *y*) is defined as follows:

(5) $$K_{nm}=\sum_{x=0}^{N-1}\sum_{y=0}^{M-1}\bar{K}n\left(x;p1,N-1\right)\;\bar{K}m\left(y;p2,M-1\right)f\left(x,y\right)$$

Krawtchouk moments are very effective local descriptors, unlike the other moment families which capture the global features of the objects they describe. This locality property is controlled by the appropriate adjustment of the *p*1, *p*2 parameters of Equation (5).

### 3.2. Watermark Embedding

**Figure 1:**
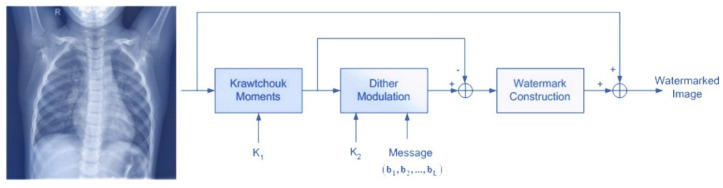
Watermark embedding.

The method we used for watermark embedding was proposed by [32] and consists of the processing modules depicted in Figure 1.

In Figure 1, the original image is the initial medical image where a L-bit length binary message is inserted by constructing the final watermarked image. A set of Krawtchouk moments is calculated according to Equation (5). In this stage, there is a key set *K_1_* that corresponds to the set of parameters *p*: (*p*1, *p*2). Dither modulation is an important methodology that integrates one signal into another one, enhances the embedding rate with minimum distortion of the original image, and increases robustness under attacking conditions. In this methodology, the Krawtchouk moments of the initial image is used as the host signal where the L-bit length binary message (*b*_1_, *b*_2_, …, *b_L_*) is inserted according to Equation (6). The modified Krawtchouk moments, which resulted from dither modulation, are used to construct the watermark information, which is added with the initial image in the last step.

(6) $${\tilde{K}}_{n_{i}m_{i}\;}=\left[\frac{K_{n_{i}m_{i}-}d_{1}\left(b_{i}\right)\;}{\Delta }\right]\Delta +d_{i}\left(b_{i}\right),\;i=1,\;\ldots ,\;L\;$$

where [.] is the rounding operator, Δ the quantization step (key *K*_2_), which is actually the embedding strength of the watermark information, and $d_{i}\left(.\right)$ the ith dither function satisfying $d_{i}\left(1\right)$= Δ/2 + $d_{i}$(0). The dither vector ($d_{1}$(0), $d_{2}$(0),…, $d_{L}$(0)) is uniformly distributed in the range [0, Δ].

### 3.3. Watermarking Adversarial Attack

Digital watermarking is a process that prevents tampering by providing authentication, content verification, and image integration. It consists of two processes. The first process is called watermark embedding, during which digital information is embedded into a multimedia product and the second one is called watermark extraction, in which the information is extracted or detected from the product. Watermarking in the medical field has numerous practical applications, including telediagnosis, teleconferencing between clinicians, and distance training of medical staff. The use of watermarking techniques guarantees the confidentiality, security of the sent data, and the integrity of the medical images. Furthermore, watermark authentication and tamper detection methods can be used to locate the source of the medical images and the falsified area, respectively. All of the above lead to the conclusion that watermarking is a crucial process and necessary in medical image analysis.

So far, we have been taking advantage of the benefits of watermarking, however, digital watermarking can garble the quality of a multimedia product such as an image. These changes may not affect human decision making, but we hypothesize that they can influence the decision of a deep learning model. In this study, we deal only with the watermark embedding part and not with the extraction part since we study the performance of the models on watermarked images. There are numerous watermarking methodologies, like other moment families [33] or transformations [34], that are applied in medical images, and these can constitute a new category of attacks. 

We experimented with a watermarking method that uses Krawtchouk moments, because image moments are one of the most important descriptors of the content of images and they are widely used in many fields of image processing. Moreover, another adversarial attack, Discrete Orthogonal Moments Exclusion of Tchebichef image moments DOME-T [35], uses moments to attack ImageNet dataset with remarkable results. Through this research, we highlight a crucial problem that has not been re-studied—that watermarking can impair the performance of the models. Watermarking is widely used in the analysis of medical images, and therefore various watermarking methodologies for the safe use of artificial intelligence in the medical field must be studied from this perspective. We name this new category of adversarial attacks as Watermarking Adversarial Attacks, or WA^2^ for short, and herein we are studying the Krawtchouk Moments based WA^2^ represented by the term KMsWA^2^.

## 4. Experimental Study

In order to investigate the performance of the proposed watermarking attack (The source code of the proposed KMsWA2 attack will be provided via the github account (https://github.com/MachineLearningVisionRG/KMsWA2, accessed on 17 April 2022) of our research group, upon acceptance of the paper), we trained three popular deep learning models, DenseNet169, DenseNet201, and MobileNetV2, which are widely used by the research community, and thus it is important to investigate their robustness. We combined all *p*1 and *p*2 values, *p*1, *p*2 ∈ [0.1, 0.2, …, 0.9], with different L-bit lengths and embedding strength a. The L-bit length ranges from 100 to 1000 with step 100. The embedding strength takes four different values, 50, 100, 200, and 300. The watermark embedding was implemented in MATLAB 2018a and the models were trained in Google Collab with Keras 2.4.3. All models were pretrained in ImageNet dataset and they were fine-tuned with Adam optimizer for 20 epochs with a learning rate of 0.0001. We also use three different attacks, FGSM, PGD, and Square Attack [36], for comparison. FGSM and PGD create samples with different models in order for them to treat as black-box attacks. For this purpose, the Adversarial Robustness Toolbox (ART) [37] for creating adversarial samples was applied. Finally, the SSIM index was calculated for the assessment of the image distortion.

### 4.1. Datasets

**Figure 2:**
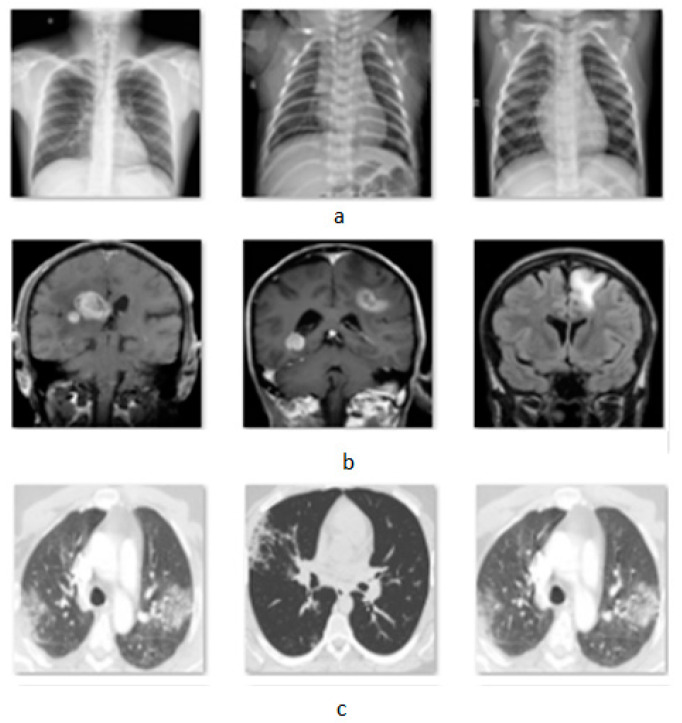
Images from three datasets, (**a**) X-rays, (**b**) MRIs, and (**c**) CT-Scans.

The attack was applied in classification problems in three different modalities. The first dataset [38] is an X-ray set from the lungs that classifies the images into three categories, normal, pneumonia, and COVID-19, containing 3840 images. The second dataset [39] consists of brain MRIs of four tumor categories with 3264 total images and the last dataset [40] is a binary classification of CT-Scans for COVID-19 and non-COVID-19 lungs, providing 2481 images. In Figure 2 is presented a sample of the used datasets.

### 4.2. Ablation Study

**Figure 3:**
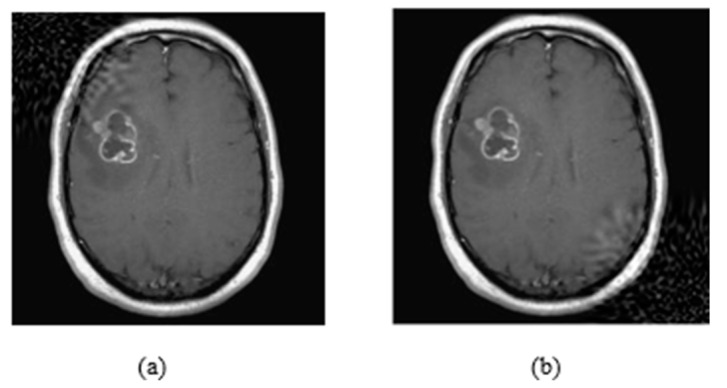
(**a**) Watermark embedding with *p*1 = 0.1 and *p*2 = 0.1, (**b**) Watermark embedding with *p*1 = 0.9 and *p*2 = 0.9.

The attack consists of three main parameters: embedding strength (a), embedding message length (L-bit), and *p* values (*p*1, *p*2). The embedding strength is an important parameter in digital watermarking because it affects the extraction of information. When the strength value is big, the extraction method is more robust, but the perturbation in images is more visible. The L-bit length concerns the size of information we insert in images. If the size is large, then the part of the image, which is perturbated, is also large. The last parameters, *p* values (*p*1, *p*2), function as coordinates of local patch of the image where the watermark is inserted (Figure 3).

**Figure 4:**
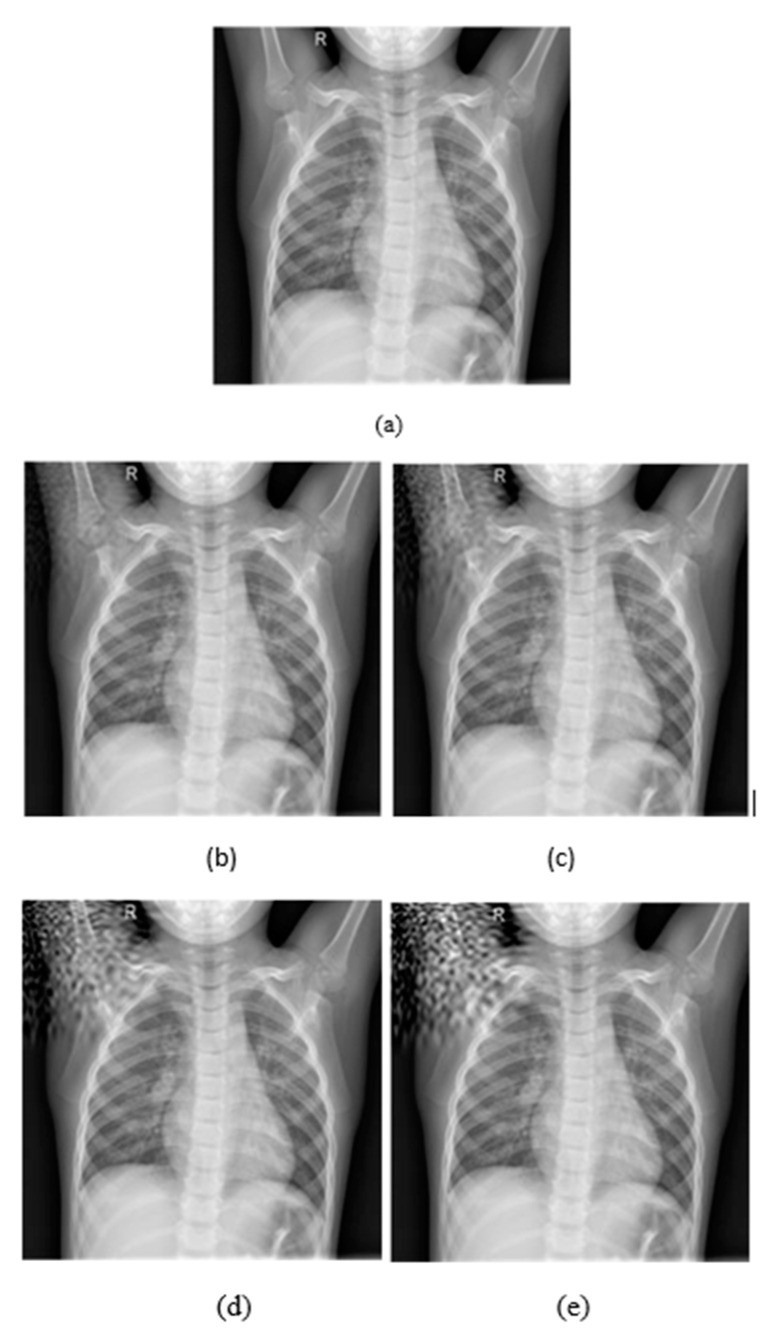
(**a**) Initial image. (**b**) Embedding strength = 50, (**c**) Embedding strength = 100, (**d**) Embedding strength = 200, (**e**) Embedding strength = 300. The rest of the parameters are L-bit = 1000, *p*1 = 0.1, *p*2 = 0.1.

**Figure 5:**
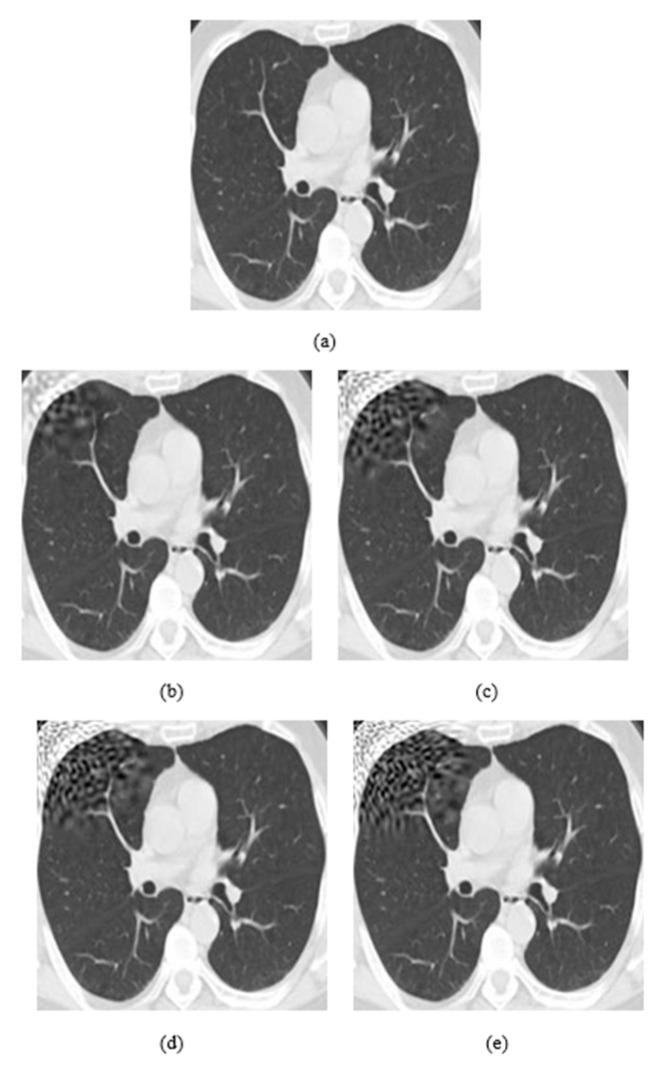
(**a**) Initial image, (**b**) L-bit = 200, (**c**) L-bit = 500, (**d**) L-bit = 800, (**e**) L-bit = 800. The rest parameters are Embedding strength = 300, *p*1 = 0.1, *p*2 = 0.1.

As it is shown in Figure 3a, the watermarking is embedded on the upper left corner, as the *p* parameters are equal to 0.1, while in (b) the watermarking was embedded on the bottom right corner because *p* values are equal to 0.9. Both *p* values range from 0.1 to 0.9 by representing all local points of the image. In Figure 4, it is presented how the embedding strength affects the distortion of an image while the other parameters are constant (L-bit = 1000, *p*1 = 0.1, *p*2 = 0.1), and in Figure 5 the perturbation is presented from L-bit length (embedding strength = 300, *p*1 = 0.1, *p*2 = 0.1). Embedding strength controls the limit of watermark information that is inserted in the image. A large embedding strength provides more robustness, but it is also more perceptible at the same time.

As it is depicted in Figure 4, increasing the embedding strength the quality of the image is getting worse and the noise becomes more perceptible and intense. On the other hand, in Figure 5 the intense of the noise is almost the same in all L-Bit lengths, but it changes the magnitude of the noise. 

**Figure 6:**
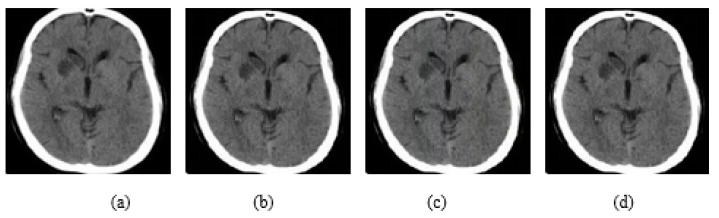
(**a**) Initial Image, (**b**) FGSM attack with ϵ = 0.01, (**c**) PGD attack with ϵ = 0.01, (**d**) Square Attack with ϵ = 0.01.

**Figure 7:**
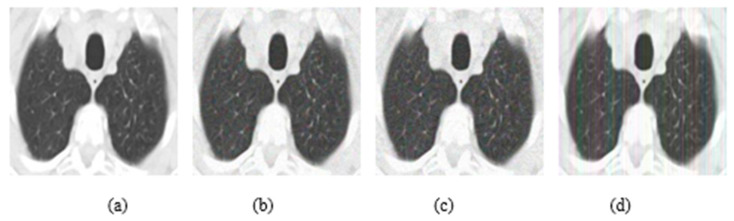
(**a**) Initial Image, (**b**) FGSM attack with ϵ = 0.07, (**c**) PGD attack with ϵ = 0.07, (**d**) Square Attack with ϵ = 0.07.

**Figure 8:**
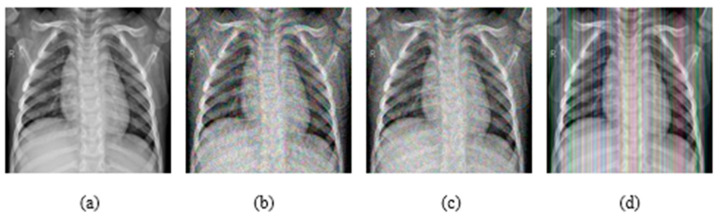
(**a**) Initial Image, (**b**) FGSM attack with ϵ = 0.15, (**c**) PGD attack with ϵ = 0.15, (**d**) Square Attack with ϵ = 0.15.

In addition, experiments were performed with FGSM, PGD, and Square Attack for ϵ values equal to 0.01, 0.03, 0.05, 0.07, 0.09, 0.12, and 0.15. In Figure 6, MRI with aforementioned attacks and ϵ = 0.01 are presented. The human eye cannot understand any difference between these images. In Figure 7, attacks with ϵ = 0.07 are depicted. Square Attack causes the biggest distortion compared to FGSM and PGD. However, small changes can be observed also in the other two attacks. In Figure 8, the ϵ value has been increased to 0.15, making the noise perceptible. 

### 4.3. Results

All possible combinations of parameters are applied in images in order to investigate, which set of parameters is more effective. As it is reasonable, big values of L-bit length and embedding strength led to greater efficiency. However, adversarial attacks should be as imperceptible as possible. That is why we experimented with all values in order to combine efficiency and imperceptibility. In Table 1, Table 2 and Table 3 the results before and after attack for X-rays Images are presented, while Table A1, Table A2 and Table A3 concern MRIs and Table A4, Table A5 and Table A6 concern CT-scans, all for the case of the three examined DL pretrained models. For each L-bit length and embedding strength, we present the most effective values of p1 and p2. Moreover, the term “original accuracy” refers to the performance of the models in non-watermarked images. Additionally, the SSIM index (it takes values between 0–1 or 0–100% in percentage) between the original and the attacked image is presented in the following tables. The lowest SSIM index was given by X-rays (0.79) with embedding strength and L-Bit length equal to 500 and 1000, respectively. The attacking performance of FGSM, PGD and Square Attack are presented in Table A7, Table A8 and Table A9 for X-rays, MRIs, and CT-Scans, respectively. The value ϵ in tables is the magnitude of perturbation for each attack. Each table shows the SSIM index and the model’s accuracy for each ϵ value. To make the text legible, Table A1, Table A2, Table A3, Table A4, Table A5, Table A6, Table A7, Table A8 and Table A9 are available for viewing in Appendix A.

## 5. Discussion

**Figure 9:**
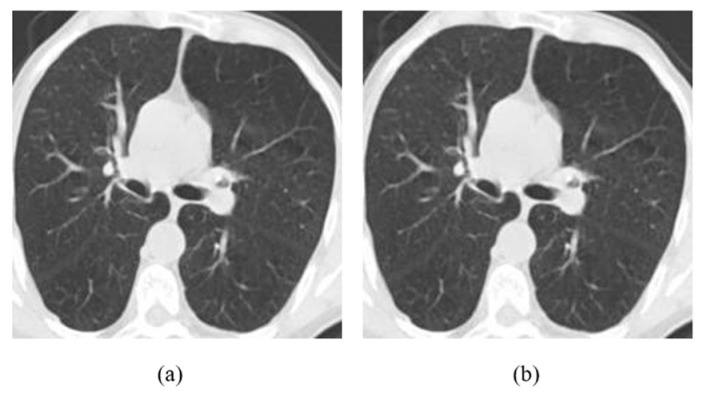
(**a**) Clean Image, (**b**) Attacked image (L-Bit = 100, Embedding strength = 50).

According to the results, CT-Scan was the least robust modality, as the accuracy of the models was reduced almost by 50%. This is very interesting, as COVID-19 detection using CT-Scans should have been the most robust problem because it has only two classes. Even with the smallest perturbation, MobileNetV2 was decreased by 12.2% in terms of accuracy (Figure 9). The CT-Scan modality should be further investigated to draw safe conclusions. The problem of brain tumor classification was the most difficult one and therefore the performance of the models, even with clean images, was low. However, the models did not lose significant accuracy with an imperceptible perturbation. On X-rays, accuracy decreases significantly when we increase the embedding strength, or we insert a lot of information.

Moreover, MobileNetV2 is the weakest model, as it loses accuracy easier than the other two models with no need for a perceptible distortion. This may be due to the fact that MobileNetV2 has fewer parameters compared to the other models. In CT-scans case, which was the weakest one, all models lost an important percentage of accuracy with the lowest values, however, the DenseNets lost their accuracy at a slower pace than MobileNetV2. Furthermore, in MRI and X-ray cases DenseNet201 and DenseNet169 need a combination of high values of embedding strength and L-Bit length to significantly reduce their accuracy. On the other hand, the accuracy of MobileNetV2 is significantly decreased when either embedding strength or L-Bit length is high. As a consequence, DenseNets variants need perceptible noise in order to decrease their accuracy. In the case of MRI, the most difficult, DenseNets variants responded very well, losing 5% of their accuracy and needing high values of embedding strength and L-Bit length, 200, and 700, respectively. The problem of classification in medical images is usually difficult because there are no important differences between the different classes. Additionally, there are cases such as X-rays from lungs in which specific points determine the decision. That is why *p*1 and *p*2 values play a significant role in the attack’s efficiency. We observe that each problem shares similar *p* values because these values show the critical points. This is an important advantage of this attack, as we can predefine the *p* values depending on the images we attack. 

**Figure 10:**
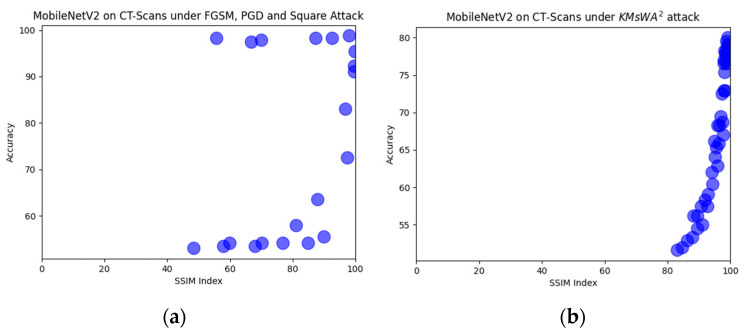
Scatter plots for MobileNetV2 in CT-Scans under (**a**) FGSM, PGD, Square Attack, and (**b**) KMsWA^2^ attack.

**Figure 11:**
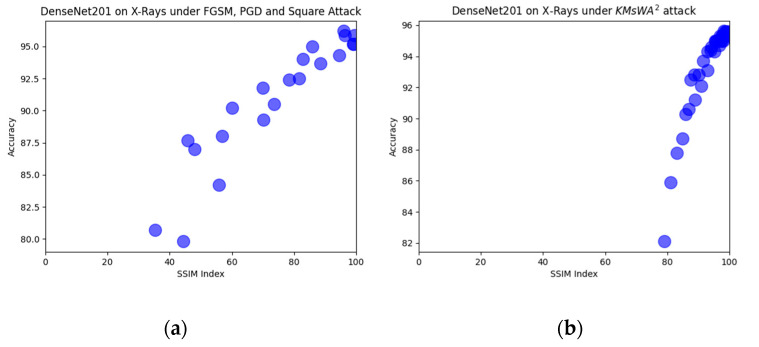
Scatter plots for DenseNet201 in X-rays under (**a**) FGSM, PGD, Square Attack, and (**b**) KMsWA^2^ attack.

**Figure 12:**
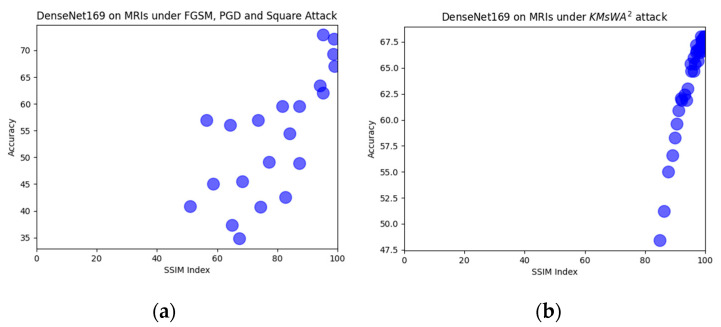
Scatter plots for DenseNet169 in MRIs under (**a**) FGSM, PGD, Square Attack, and (**b**) KMsWA^2^ attack.

The comparison with the other attacks shows that there is not a clear winner. In CT-Scan modality, the proposed attack achieved the greatest accuracy degradation in all models by presenting a much better SSIM index. In X-rays there are cases in which the other three attacks are more effective but with worse SSIM index. For instance, PGD with ϵ = 0.15 dropped the accuracy to 79.8% with SSIM = 44.3%, while the proposed attack at 82% with SSIM = 80%. The proposed KMsWA^2^ attack shows a high SSIM index even with the high values of the embedding strength, and the L-Bit length is shown in Figure 10, Figure 11 and Figure 12. This is due to the fact that watermarking applied only to the *p* values and not to the whole image. The other attacks create adversarial noise on the whole image, destroying its quality.

In Figure 10, Figure 11 and Figure 12, six representative scatter plots for the three image modalities are presented, showing that the proposed KMsWA^2^ attack achieves the same or better performance degradation with significantly higher SSIM index. In Figure 10a, Figure 11a, and Figure 12a, the dots are scattered from top right to bottom and left, indicating that the reduction in the accuracy is achieved only with low SSIM index, while Figure 10b, Figure 11b, and Figure 12b present a vertical direction, which means that the proposed KMsWA^2^ attack drops the accuracy without dropping much SSIM index. These results constitute evidence that watermarking can be considered as an adversarial attack for the images and thus the research community should study this phenomenon deeply, otherwise the watermarking methods will be inhibitors to the computer vision applications in medical image analysis.

## 6. Conclusions

In this study, we proposed a black-box adversarial attack for medical images using a moment-based watermarking methodology. We experimented with three different modalities, X-rays, MRIs, and CT-Scans, achieving performance degradation up to 41% to the model, proving that digital watermarking may act as a trojan because it is usually used for the patient’s privacy and safety. However, we showed that even with the least insertion of information or the smallest embedding strength, the performance can be reduced. Moreover, the experiments revealed that the proposed attack is competitive to the established adversarial attacks since it affects the accuracy of the deep learning models in an imperceptible way without being perceived by human eyes. In addition, defending against this attack is not an easy process because the images are distorted locally, and a huge number of images must be created to apply adversarial learning. DenseNets models were the most robust, while MobileNetV2 was the weakest and CT-scans was the most vulnerable modality. As future work, we would like to experiment with more watermarking methodologies as well as more moment families following the same scheme proposed herein and also to examine other popular medical image watermarking techniques, e.g., based on wavelets. Moreover, we are planning to investigate if adversarial learning is able to alleviate the effects of watermarking attacks.

## Figures and Tables

**Table 1:** KMsWA^2^ attack on MobileNetV2 in X-rays dataset.

X-rays − MobileNetV2 − Original Accuracy = 96.8%												
L-Bits	Embed. Strength = 50			Embed. Strength = 100			Embed. Strength = 200			Embed. Strength = 300		
	SSIM (%)	*p*1, *p*2	Acc. (%)	SSIM (%)	*p*1, *p*2	Acc. (%)	SSIM (%)	*p*1, *p*2	Acc. (%)	SSIM (%)	*p*1, *p*2	Acc. (%)
**100**	99.4	0.8, 0.1	95.3	99.2	0.9, 0.6	94.3	98.4	0.8, 0.6	93.1	97.5	0.8, 0.4	92.1
**200**	99.3	0.9, 0.6	95.0	98.7	0.8, 0.6	93.7	97.0	0.8, 0.4	91.8	95.2	0.8, 0.6	91.8
**300**	99.0	0.9, 0.6	95.0	98.2	0.8, 0.5	93.4	95.6	0.8, 0.5	90.3	93.0	0.8, 0.5	90.6
**400**	98.9	0.9, 0.6	94.3	97.6	0.8, 0.4	93.1	94.2	0.8, 0.4	89.3	90.9	0.8, 0.4	88.7
**500**	98.7	0.8, 0.5	93.7	97.1	0.8, 0.4	92.8	92.9	0.8, 0.4	89.0	88.9	0.7, 0.4	89.3
**600**	98.5	0.8, 0.5	94.0	96.5	0.7, 0.4	92.1	91.5	0.7, 0.5	87.5	86.9	0.7, 0.5	87.8
**700**	98.3	0.8, 0.5	93.7	95.9	0.7, 0.5	91.2	90.2	0.7, 0.3	86.8	84.9	0.7, 0.5	86.8
**800**	98.1	0.8, 0.5	93.4	95.3	0.7, 0.5	90.0	88.8	0.7, 0.5	87.5	83.0	0.7, 0.5	84.3
**900**	97.9	0.7, 0.5	93.1	96.7	0.7, 0.6	89.3	87.4	0.7, 0.5	83.4	81.0	0.7, 0.5	79.0
**1000**	97.6	0.7, 0.5	93.1	94.0	0.7, 0.6	88.1	85.9	0.7, 0.5	82.1	79.0	0.7, 0.5	78.7

**Table 2:** KMsWA^2^ attack on DenseNet201 in X-rays.

X-rays − DenseNet201 − Original Accuracy = 96.2%												
L-Bits	Embed. Strength = 50			Embed. Strength = 100			Embed. Strength = 200			Embed. Strength = 300		
	SSIM (%)	*p*1, *p*2	Acc. (%)	SSIM (%)	*p*1, *p*2	Acc. (%)	SSIM (%)	*p*1, *p*2	Acc. (%)	SSIM (%)	*p*1, *p*2	Acc. (%)
**100**	99.4	0.8, 0.8	95.5	99.2	0.8, 0.4	95.3	98.4	0.8, 0.7	95.3	97.5	0.4, 0.6	95.0
**200**	99.3	0.8, 0.8	95.6	98.7	0.9, 0.1	95.3	97.0	0.1, 0.7	95.3	95.2	0.8, 0.6	94.3
**300**	99.0	0.8, 0.1	95.3	98.2	0.3, 0.5	95.6	95.6	0.8, 0.7	95.0	93.0	0.5, 0.5	93.1
**400**	98.9	0.9, 0.5	95.6	97.6	0.1, 0.7	95.3	94.2	0.1, 0.9	94.6	90.9	0.4, 0.5	92.1
**500**	98.7	0.1, 0.8	95.3	97.1	0.8, 0.6	95.0	92.9	0.1, 0.7	94.3	88.9	0.5, 0.5	91.2
**600**	98.5	0.8, 0.1	95.6	96.5	0.1, 0.7	95.0	91.5	0.4, 0.6	93.7	86.9	0.6, 0.7	90.6
**700**	98.3	0.8, 0.5	95.3	95.9	0.8, 0.9	95.0	90.2	0.3, 0.5	92.8	84.9	0.6, 0.8	88.7
**800**	98.1	0.1, 0.2	95.3	95.3	0.5, 0.1	95.0	88.8	0.4, 0.5	92.8	83.0	0.4, 0.6	87.8
**900**	97.9	0.9, 0.5	95.0	96.7	0.1, 0.8	94.7	87.4	0.6, 0.6	92.5	81.0	0.4, 0.7	85.9
**1000**	97.6	0.1, 0.8	95.3	94.0	0.4, 0.3	94.4	85.9	0.4, 0.5	90.3	79.0	0.4, 0.5	82.1

**Table 3:** KMsWA^2^ attack on DenseNet169 in X-rays.

X-rays − DenseNet169 − Original Accuracy = 95.9%												
L-Bits	Embed. Strength = 50			Embed. Strength = 100			Embed. Strength = 200			Embed. Strength = 300		
	SSIM (%)	*p*1, *p*2	Acc. (%)	SSIM (%)	*p*1, *p*2	Acc. (%)	SSIM (%)	*p*1, *p*2	Acc. (%)	SSIM (%)	*p*1, *p*2	Acc. (%)
**100**	99.4	0.8, 0.4	95.3	99.2	0.9, 0.3	95.0	98.4	0.8, 0.5	94.0	97.5	0.8, 0.7	94.3
**200**	99.3	0.8, 0.4	95.0	98.7	0.1, 0.8	95.0	97.0	0.8, 0.5	93.7	95.2	0.8, 0.5	91.2
**300**	99.0	0.8, 0.4	95.3	98.2	0.1, 0.5	94.3	95.6	0.7, 0.5	92.5	93.0	0.8, 0.5	89.3
**400**	98.9	0.8, 0.8	95.0	97.6	0.8, 0.5	94.0	94.2	0.7, 0.5	91.5	90.9	0.8, 0.5	89.3
**500**	98.7	0.8, 0.5	94.6	97.1	0.7, 0.5	93.7	92.9	0.7, 0.5	90.3	88.9	0.7, 0.4	88.4
**600**	98.5	0.8, 0.4	95.0	96.5	0.7, 0.5	94.0	91.5	0.7, 0.4	89.6	86.9	0.6, 0.5	85.9
**700**	98.3	0.9, 0.3	94.6	95.9	0.7, 0.4	93.4	90.2	0.7, 0.3	88.4	84.9	0.6, 0.5	85.3
**800**	98.2	0.9, 0.3	95.0	95.3	0.2, 0.3	93.7	88.8	0.7, 0.5	87.8	83.0	0.6, 0.5	84.0
**900**	97.9	0.9, 0.4	94.6	96.7	0.7, 0.4	91.5	87.4	0.7, 0.5	87.1	81.0	0.6, 0.5	80.6
**1000**	97.6	0.8, 0.6	94.6	94.0	0.7, 0.4	91.5	85.9	0.6, 0.5	85.0	79.0	0.6, 0.5	80.3

## Data Availability

https://github.com/MachineLearningVisionRG/KMsWA2 (accessed on 17 April 2022).

## Appendix A

**Table A1:** KMsWA^2^ attack on MobileNetV2 in MRIs.

MRIs − MobileNetV2 − Original Accuracy = 77.6%												
L-Bits	Embed. Strength = 50			Embed. Strength = 100			Embed. Strength = 200			Embed. Strength = 300		
	SSIM (%)	*p*1, *p2*	Acc. (%)	SSIM (%)	*p*1, *p*2	Acc. (%)	SSIM (%)	*p*1, *p*2	Acc. (%)	SSIM (%)	*p*1, *p*2	Acc. (%)
**100**	99.9	0.4, 0.4	76.1	99.8	0.1, 0.6	76.6	99.2	0.6, 0.4	75.6	98.6	0.6, 0.6	73.6
**200**	99.8	0.2, 0.8	76.6	99.5	0.1, 0.6	75.8	98.3	0.6, 0.4	74.1	97.0	0.5, 0.7	70.0
**300**	99.7	0.2, 0.7	76.1	99.1	0.5, 0.4	75.3	97.2	0.6, 0.6	71.8	95.3	0.5, 0.7	66.5
**400**	99.6	0.4, 0.5	75.9	98.7	0.3, 0.4	74.8	96.2	0.6, 0.7	71.0	93.7	0.5, 0.7	65.4
**500**	99.5	0.7, 0.4	76.1	98.3	0.6, 0.5	73.3	95.2	0.5, 0.8	68.5	92,0	0.4, 0.7	63.2
**600**	99.4	0.5, 0.6	75.6	97.9	0.6, 0.5	73.8	94.2	0.5, 0.5	66.0	90.6	0.4, 0.5	60.9
**700**	99.2	0.5, 0.6	75.1	97.5	0.5, 0.6	73.1	93.2	0.5, 0.5	66.7	89.1	0.4, 0.5	60.4
**800**	99.0	0.5, 0.6	74.8	97.0	0.6, 0.5	72.6	92.1	0.4, 0.5	65.4	87.7	0.4, 0.5	58.6
**900**	98.9	0.5, 0.6	74.1	96.6	0.4, 0.4	71.3	91.1	0.4, 0.5	63.2	86.2	0.5, 0.5	56.3
**1000**	98.8	0.5, 0.6	74.5	98.8	0.4, 0.5	70.3	90.0	0.4, 0.5	62.6	84.9	0.4, 0.5	54.8

**Table A2:** KMsWA^2^ attack on DenseNet201 in MRIs.

MRIs − DenseNet201 − Original Accuracy = 71.3%												
L-Bits	Embed. Strength = 50			Embed. Strength = 100			Embed. Strength = 200			Embed. Strength = 300		
	SSIM (%)	*p*1, *p*2	Acc. (%)	SSIM (%)	*p*1, *p*2	Acc. (%)	SSIM (%)	*p*1, *p*2	Acc. (%)	SSIM (%)	*p*1, *p*2	Acc. (%)
**100**	99.9	0.2, 0.2	69.8	99.8	0.7, 0.1	69.8	99.2	0.5, 0.8	69.5	98.6	0.7, 0.1	69.0
**200**	99.8	0.3, 0.8	69.8	99.5	0.7, 0.1	69.5	98.3	0.7, 0.5	69.0	97.0	0.4, 0.4	68.3
**300**	99.7	0.3, 0.2	69.5	99.1	0.7, 0.1	69.5	97.2	0.6, 0.9	68.2	95.3	0.6, 0.1	67.2
**400**	99.6	0.8, 0.7	69.5	98.7	0.7, 0.1	69.0	96.2	0.4, 0.6	68.5	93.7	0.6, 0.6	65.7
**500**	99.5	0.9, 0.8	69.8	98.3	0.6, 0.9	69.0	95.2	0.6, 0.9	68.0	92,0	0.6, 0.6	64.4
**600**	99.4	0.6, 0.9	69.5	97.9	0.5, 0.9	69.0	94.2	0.7, 0.7	66.7	90.6	0.4, 0.4	64.2
**700**	99.2	0.7, 0.9	69.5	97.5	0.6, 0.9	69.2	93.2	0.4, 0.5	64.5	89.1	0.4, 0.5	61.7
**800**	99.0	0.1, 0.2	69.5	97.0	0.9, 0.8	68.5	92.1	0.4, 0.5	64.2	87.7	0.5, 0.5	57.1
**900**	98.9	0.6, 0.9	69.0	96.6	0.9, 0.8	68.0	91.1	0.4, 0.5	63.4	86.2	0.4, 0.5	56.6
**1000**	98.8	0.6, 0.9	69.0	98.8	0.9, 0.8	68.7	90.0	0.4, 0.5	61.2	84.9	0.5, 0.5	55.0

**Table A3:** KMsWA^2^ attacks on DenseNet169 in MRIs.

MRIs − DenseNet169 − Original Accuracy = 69.54%												
L-Bits	Embed. Strength = 50			Embed. Strength = 100			Embed. Strength = 200			Embed. Strength = 300		
	SSIM (%)	*p*1, *p*2	Acc. (%)	SSIM (%)	*p*1, *p*2	Acc. (%)	SSIM (%)	*p*1, *p*2	Acc. (%)	SSIM (%)	*p*1, *p*2	Acc. (%)
**100**	99.9	0.5, 0.6	67.5	99.8	0.8, 0.5	68.0	99.2	0.9, 0.4	66.7	98.6	0.8, 0.2	68.0
**200**	99.8	0.7, 0.5	67.0	99.5	0.8, 0.4	68.0	98.3	0.9, 0.4	66.5	97.0	0.5, 0.6	67.2
**300**	99.7	0.7, 0.5	67.0	99.1	0.3, 0.5	67.5	97.2	0.2, 0.4	66.7	95.3	0.2, 0.5	64.7
**400**	99.6	0.8, 0.2	67.0	98.7	0.3, 0.5	67.7	96.2	0.4, 0.5	66.0	93.7	0.2, 0.5	61.9
**500**	99.5	0.9, 0.4	67.0	98.3	0.9, 0.5	67.0	95.2	0.4, 0.5	65.4	92,0	0.4, 0.4	62.1
**600**	99.4	0.7, 0.5	67.5	97.9	0.9, 0.5	66.7	94.2	0.3, 0.4	63.0	90.6	0.4, 0.4	59.6
**700**	99.2	0.9, 0.4	67.5	97.5	0.6, 0.6	65.7	93.2	0.4, 0.4	62.4	89.1	0.4, 0.4	56.6
**800**	99.0	0.9, 0.6	67.7	97.0	0.6, 0.6	66.5	92.1	0.3, 0.5	61.9	87.7	0.4, 0.4	55.0
**900**	98.9	0.9, 0.6	67.7	96.6	0.5, 0.6	65.4	91.1	0.4, 0.5	60.9	86.2	0.5, 0.5	51.2
**1000**	98.8	0.9, 0.6	67.2	98.8	0.5, 0.6	64.7	90.0	0.4, 0.5	58.3	84.9	0.5, 0.5	48.4

**Table A4:** KMsWA^2^ attacks on MobileNetV2 in CT-Scans.

CT-Scans − MobileNetV2 − Original Accuracy = 92.2%												
L-Bits	Embed. Strength = 50			Embed. Strength = 100			Embed. Strength = 200			Embed. Strength = 300		
	SSIM (%)	*p*1, *p*2	Acc. (%)	SSIM (%)	*p*1, *p*2	Acc. (%)	SSIM (%)	*p*1, *p*2	Acc. (%)	SSIM (%)	*p*1, *p*2	Acc. (%)
**100**	99.2	0.7, 0.4	80.0	99.0	0.2, 0.8	77.9	98.4	0.8, 0.7	72.9	97.7	0.8, 0.8	67.0
**200**	99.1	0.2, 0.9	79.1	98.7	0.8, 0.8	76.6	97.4	0.7, 0.6	68.7	95.9	0.6, 0.7	62.9
**300**	99.0	0.2, 0.9	78.7	98.2	0.8, 0.5	75.4	96.2	0.9, 0.6	65.8	94.2	0.8, 0.6	60.4
**400**	98.8	0.9, 0.6	79.5	97.8	0.8, 0.5	72.9	95.1	0.9, 0.5	64.1	92.6	0.9, 0.4	57.5
**500**	98.7	0.8, 0.5	78.3	97.3	0.8, 0.5	72.5	94.0	0.8, 0.5	62.0	91.0	0.9, 0.5	55.0
**600**	98.5	0.7, 0.5	77.0	96.8	0.8, 0.5	69.5	92.9	0.9, 0.4	59.1	89.4	0.9, 0.5	54.5
**700**	98.3	0.8, 0.5	77.9	96.4	0.9, 0.6	68.3	91.8	0.9, 0.5	58.3	87.8	0.9, 0.5	53.3
**800**	98.2	0.9, 0.6	78.3	95.9	0.9, 0.6	68.3	90.7	0.9, 0.6	57.5	86.3	0.9, 0.5	52.9
**900**	98.0	0.5, 0.6	77.0	95.4	0.9, 0.6	65.3	89.5	0.9, 0.6	56.2	84.6	0.9, 0.5	52.0
**1000**	97.8	0.7, 0.5	76.6	94.9	0.9, 0.6	66.2	88.3	0.9, 0.5	56.2	83.0	0.9, 0.5	51.6

**Table A5:** KMsWA^2^ attacks on DenseNet201 in CT-Scans.

CT-Scans − DenseNet201 − Original Accuracy = 96.6%												
L-Bits	Embed. Strength = 50			Embed. Strength = 100			Embed. Strength = 200			Embed. Strength = 300		
	SSIM (%)	*p*1, *p*2	Acc. (%)	SSIM(%)	*p*1, *p*2	Acc. (%)	SSIM (%)	*p*1, *p*2	Acc. (%)	SSIM (%)	*p*1, *p*2	Acc. (%)
**100**	99.2	0.6, 0.8	89.1	99.0	0.3, 0.9	87.9	98.4	0.4, 0.9	87.0	97.7	0.7, 0.6	83.7
**200**	99.1	0.3, 0.2	89.1	98.7	0.3, 0.9	87.9	97.4	0.6, 0.7	84.5	95.9	0.6, 0.7	75.8
**300**	99.0	0.9, 0.9	88.3	98.2	0.3, 0.9	87.5	96.2	0.6, 0.3	81.2	94.2	0.7, 0.7	71.6
**400**	98.8	0.9, 0.2	88.7	97.8	0.3, 0.9	88.3	95.1	0.6, 0.3	79.5	92.6	0.6, 0.5	70.8
**500**	98.7	0.5, 0.1	89.1	97.3	0.4, 0.6	87.5	94.0	0.6, 0.4	76.6	91.0	0.7, 0.4	65.8
**600**	98.5	0.7, 0.2	88.3	96.8	0.4, 0.6	87.0	92.9	0.7, 0.5	76.6	89.4	0.6, 0.5	65.8
**700**	98.3	0.8, 0.9	88.7	96.4	0.4, 0.6	86.2	91.8	0.7, 0.5	75.8	87.8	0.6, 0.6	65.8
**800**	98.2	0.7, 0.4	88.7	95.9	0.4, 0.6	85.8	90.7	0.7, 0.5	75.0	86.3	0.7, 0.5	65.4
**900**	98.0	0.5, 0.9	88.3	95.4	0.4, 0.6	85.8	89.5	0.7, 0.5	75.0	84.6	0.6, 0.5	65.8
**1000**	97.8	0.9, 0.6	88.3	94.9	0.4, 0.6	86.2	88.3	0.7, 0.5	75.0	83.0	0.9, 0.5	65.8

**Table A6:** KMsWA^2^ attacks on DenseNet169 in CT-Scans.

CT-Scans − DenseNet169 − Original Accuracy = 95.8%												
L-Bits	Embed. Strength = 50			Embed. Strength = 100			Embed. Strength = 200			Embed. Strength = 300		
	SSIM (%)	*p*1, *p*2	Acc. (%)	SSIM (%)	*p*1, *p*2	Acc. (%)	SSIM (%)	*p*1, *p*2	Acc. (%)	SSIM (%)	*p*1, *p*2	Acc. (%)
**100**	99.2	0.4, 0.2	89.5	99.0	0.2, 0.3	87.9	98.4	0.4, 0.8	82.9	97.7	0.3, 0.3	77.0
**200**	99.1	0.7, 0.2	89.5	98.7	0.3, 0.3	87.5	97.4	0.3, 0.1	79.5	95.9	0.3, 0.3	73.7
**300**	99.0	0.7, 0.2	89.5	98.2	0.3, 0.1	86.6	96.2	0.4, 0.1	79.1	94.2	0.4, 0.3	70.0
**400**	98.8	0.7, 0.2	90.0	97.8	0.3, 0.1	87.0	95.1	0.4, 0.2	78.7	92.6	0.4, 0.3	70.0
**500**	98.7	0.5, 0.8	90.0	97.3	0.4, 0.1	86.2	94.0	0.4, 0.1	80.0	91.0	0.4, 0.4	69.1
**600**	98.5	0.7, 0.2	89.5	96.8	0.3, 0.1	85.4	92.9	0.1, 0.4	75.8	89.4	0.4, 0.5	67.0
**700**	98.3	0.9, 0.9	89.5	96.4	0.1, 0.4	86.2	91.8	0.1, 0.4	76.6	87.8	0.4, 0.5	65.8
**800**	98.2	0.1, 0.3	89.1	95.9	0.1, 0.3	86.6	90.7	0.1, 0.4	75.8	86.3	0.4, 0.5	67.0
**900**	98.0	0.9, 0.9	89.5	95.4	0.1, 0.5	84.5	89.5	0.1, 0.4	74.1	84.6	0.1, 0.5	64.5
**1000**	97.8	0.9, 0.9	89.1	94.9	0.1, 0.3	85.0	88.3	0.1, 0.5	72.0	83.0	0.1, 0.5	65.0

**Table A7:** SSIM index and performance for each attack in X-rays.

X-rays				
Attack	SSIM (%)	Acc. (%)		
		MobileNetV2	DenseNet201	DenseNet169
FGSM ϵ = 0.01	98.9	95.3	95.2	96.2
FGSM ϵ = 0.03	94.6	86.1	94.3	95.0
FGSM ϵ = 0.05	82.8	65.9	94.0	80.4
FGSM ϵ = 0.07	73.6	54.9	90.5	71.3
FGSM ϵ = 0.09	60.1	42.9	90.2	65.3
FGSM ϵ = 0.12	45.7	36.0	87.7	62.1
FGSM ϵ = 0.15	35.3	36.9	80.7	60.2
PGD ϵ = 0.01	99.2	95.9	95.2	95.6
PGD ϵ = 0.03	96.3	90.9	95.9	95.3
PGD ϵ = 0.05	88.5	70.3	93.7	87.0
PGD ϵ = 0.07	81.7	55.5	92.5	75.7
PGD ϵ = 0.09	70.1	41.6	89.3	68.5
PGD ϵ = 0.12	55.9	34.0	84.2	62.8
PGD ϵ = 0.15	44.3	33.4	79.8	60.0
Sq. At ϵ = 0.01	99.3	97.5	95.9	95.9
Sq. At ϵ = 0.03	95.9	97.1	96.2	95.9
Sq. At ϵ = 0.05	85.9	82.0	95.0	93.4
Sq. At ϵ = 0.07	78.5	65.0	92.4	89.3
Sq. At ϵ = 0.09	70.0	54.9	91.8	85.5
Sq. At ϵ = 0.12	56.9	53.3	88.0	83.6
Sq. At ϵ = 0.15	48.0	53.0	87.0	79.8

**Table A8:** SSIM index and performance for each attack in MRIs.

MRIs				
Attack	SSIM (%)	Acc. (%)		
		MobileNetV2	DenseNet201	DenseNet169
FGSM ϵ = 0.01	98.5	72.9	71.9	69.3
FGSM ϵ = 0.03	94.1	60.1	67.3	63.4
FGSM ϵ = 0.05	84.0	48.4	51.9	54.5
FGSM ϵ = 0.07	77.3	41.2	45.0	49.1
FGSM ϵ = 0.09	68.3	33.5	38.4	45.5
FGSM ϵ = 0.12	58.7	28.4	37.6	45.0
FGSM ϵ = 0.15	51.1	26.6	37.0	40.9
PGD ϵ = 0.01	98.8	76.5	74.6	72.1
PGD ϵ = 0.03	95.2	70.3	74.2	72.9
PGD ϵ = 0.05	87.2	65.5	65.7	59.6
PGD ϵ = 0.07	81.6	63.2	60.6	59.6
PGD ϵ = 0.09	73.6	56.3	54.5	57.0
PGD ϵ = 0.12	64.3	50.7	49.9	56.0
PGD ϵ = 0.15	56.5	47.0	49.1	57.0
Sq. At ϵ = 0.01	99.0	75.7	72.4	67.0
Sq. At ϵ = 0.03	95.2	65.5	69.3	62.1
Sq. At ϵ = 0.05	87.3	52.9	51.0	48.9
Sq. At ϵ = 0.07	82.7	42.5	41.5	42..5
Sq. At ϵ = 0.09	74.4	37..9	37.0	40.7
Sq. At ϵ = 0.12	67.4	34.0	33.8	34.8
Sq. At ϵ = 0.15	65.0	35.6	35.6	37.3

**Table A9:** SSIM index and performance for each attack in CT-Scans.

CT-Scans				
Attack	SSIM (%)	Acc. (%)		
		MobileNetV2	DenseNet201	DenseNet169
FGSM ϵ = 0.01	99.6	92.3	92.0	93.2
FGSM ϵ = 0.03	96.7	83.0	88.6	94.0
FGSM ϵ = 0.05	88.0	63.6	82.6	84.3
FGSM ϵ = 0.07	81.0	58.0	78.8	81.4
FGSM ϵ = 0.09	70.2	54.2	78.0	80.5
FGSM ϵ = 0.12	57.8	53.4	78.8	78.0
FGSM ϵ = 0.15	48.3	53.0	80.0	78.0
PGD ϵ = 0.01	99.8	95.4	90.4	95.8
PGD ϵ = 0.03	98.0	98.8	86.7	97.5
PGD ϵ = 0.05	92.6	98.3	70.8	91.2
PGD ϵ = 0.07	87.3	98.3	65.0	79.6
PGD ϵ = 0.09	70.0	97.9	61.7	75.0
PGD ϵ = 0.12	66.7	97.5	62.5	79.2
PGD ϵ = 0.15	55.7	98.3	62.5	76.7
Sq. At ϵ = 0.01	99.6	91.1	91.5	93.2
Sq. At ϵ = 0.03	97.3	72.5	90.7	91.5
Sq. At ϵ = 0.05	89.9	55.5	77.1	80.0
Sq. At ϵ = 0.07	84.8	54.2	69.5	72.4
Sq. At ϵ = 0.09	76.8	54.2	61.9	60.6
Sq. At ϵ = 0.12	68.0	53.4	53.8	53.8
Sq. At ϵ = 0.15	59.8	54.2	58.9	55.0

## References

[1] Geiger A., Lenz P., Stiller C., Urtasun R. (2013). Vision meets robotics: The KITTI dataset. Int. J. Robot. Res..

[2] Apostolidis K.D., Polyzos T., Grigoriadis I., Papakostas G.A. (2021). Evaluating Convolutional Neural Networks for No-Reference Image Quality Assessment. Proceedings of the 2021 4th International Conference on Signal Processing and Information Security (ICSPIS).

[3] Apostolidis K., Amanatidis P., Papakostas G. (2020). Performance Evaluation of Convolutional Neural Networks for Gait Recognition. Proceedings of the 24th Pan-Hellenic Conference on Informatics.

[4] Filippidou F.P., Papakostas G.A. (2020). Single Sample Face Recognition Using Convolutional Neural Networks for Automated Attendance Systems. Proceedings of the 2020 Fourth International Conference On Intelligent Computing in Data Sciences (ICDS).

[5] Shankar K., Zhang Y., Liu Y., Wu L., Chen C.-H. (2020). Hyperparameter Tuning Deep Learning for Diabetic Retinopathy Fundus Image Classification. IEEE Access.

[6] Fang R., Cai C. (2021). Computer vision based obstacle detection and target tracking for autonomous vehicles. MATEC Web Conf..

[7] Maliamanis T., Papakostas G.A. (2021). Machine Learning Vulnerability in Medical Imaging. Machine Learning, Big Data, and IoT for Medical Informatics.

[8] Goodfellow I.J., Shlens J., Szegedy C. (2015). Explaining and Harnessing Adversarial Examples. arXiv.

[9] Madry A., Makelov A., Schmidt L., Tsipras D., Vladu A. (2019). Towards Deep Learning Models Resistant to Adversarial Attacks. arXiv.

[10] Papernot N., McDaniel P., Jha S., Fredrikson M., Celik Z.B., Swami A. (2015). The Limitations of Deep Learning in Adversarial Settings. arXiv.

[11] Carlini N., Wagner D. (2017). Towards Evaluating the Robustness of Neural Networks. arXiv.

[12] Xu W., Evans D., Qi Y. (2018). Feature Squeezing: Detecting Adversarial Examples in Deep Neural Networks. arXiv.

[13] Apostolidis K.D., Papakostas G.A. (2021). A Survey on Adversarial Deep Learning Robustness in Medical Image Analysis. Electronics.

[14] Kuang L.-Q., Zhang Y., Han X. A Medical Image Authentication System Based on Reversible Digital Watermarking. Proceedings of the 2009 First International Conference on Information Science and Engineering.

[15] Yılmaz I., Baza M., Amer R., Rasheed A., Amsaad F., Morsi R. (2021). On the Assessment of Robustness of Telemedicine Applications against Adversarial Machine Learning Attacks. Proceedings of the International Conference on Industrial, Engineering and Other Applications of Applied Intelligent Systems.

[16] Pal B., Gupta D., Rashed-Al-Mahfuz M., Alyami S.A., Moni M.A. (2021). Vulnerability in Deep Transfer Learning Models to Adversarial Fast Gradient Sign Attack for COVID-19 Prediction from Chest Radiography Images. Appl. Sci..

[17] Paul R., Schabath M., Gillies R., Hall L., Goldgof D. (2020). Mitigating Adversarial Attacks on Medical Image Understanding Systems. Proceedings of the 2020 IEEE 17th International Symposium on Biomedical Imaging (ISBI).

[18] Huq A., Pervin M.T. (2020). Analysis of Adversarial Attacks on Skin Cancer Recognition. Proceedings of the 2020 International Conference on Data Science and Its Applications (ICoDSA).

[19] Ma X., Niu Y., Gu L., Wang Y., Zhao Y., Bailey J., Lu F. (2020). Understanding Adversarial Attacks on Deep Learning Based Medical Image Analysis Systems. arXiv.

[20] Ozbulak U., Van Messem A., De Neve W. (2019). Impact of Adversarial Examples on Deep Learning Models for Biomedical Image Segmentation. arXiv.

[21] Chen L., Bentley P., Mori K., Misawa K., Fujiwara M., Rueckert D. (2019). Intelligent image synthesis to attack a segmentation CNN using adversarial learning. arXiv.

[22] Tian B., Guo Q., Juefei-Xu F., Chan W.L., Cheng Y., Li X., Xie X., Qin S. (2021). Bias Field Poses a Threat to DNN-based X-ray Recognition. arXiv.

[23] Kugler D. (2021). Physical Attacks in Dermoscopy: An Evaluation of Robustness for clinical Deep-Learning. J. Mach. Learn. Biomed. Imaging.

[24] Shao M., Zhang G., Zuo W., Meng D. (2021). Target attack on biomedical image segmentation model based on multi-scale gradients. Inf. Sci..

[25] Yao Q., He Z., Lin Y., Ma K., Zheng Y., Zhou S.K. (2021). A Hierarchical Feature Constraint to Camouflage Medical Adversarial Attacks. arXiv.

[26] Papakostas G.A., Karakasis E.G., Koulouriotis D.E. (2010). Novel moment invariants for improved classification performance in computer vision applications. Pattern Recognit..

[27] Papakostas G.A., Boutalis Y.S., Karras D.A., Mertzios B.G. (2007). A new class of Zernike moments for computer vision applications. Inf. Sci..

[28] Kalampokas T., Papakostas G.A. (2021). Moment Transform-Based Compressive Sensing in Image. arXiv.

[29] Papakostas G.A., Boutalis Y.S., Karras D.A., Mertzios B.G. (2010). Efficient computation of Zernike and Pseudo-Zernike moments for pattern classification applications. Pattern Recognit. Image Anal..

[30] Mukundan R., Ong S.H., Lee P.A. (2001). Image analysis by Tchebichef moments. IEEE Trans. Image Process..

[31] Yap P.-T., Paramesran R., Ong S.-H. (2003). Image analysis by krawtchouk moments. IEEE Trans. Image Process..

[32] Papakostas G.A., Tsougenis E.D., Koulouriotis D.E. (2014). Moment-based local image watermarking via genetic optimization. Appl. Math. Comput..

[33] Yang C., Li J., Bhatti U.A., Liu J., Ma J., Huang M. (2021). Robust Zero Watermarking Algorithm for Medical Images Based on Zernike-DCT. Secur. Commun. Netw..

[34] Thakkar F.N., Srivastava V.K. (2017). A blind medical image watermarking: DWT-SVD based robust and secure approach for telemedicine applications. Multimed. Tools Appl..

[35] Maliamanis T., Papakostas G.A. DOME-T: Adversarial computer vision attack on deep learning models based on Tchebichef image moments. Proceedings of the Thirteenth International Conference on Machine Vision.

[36] Andriushchenko M., Croce F., Flammarion N., Hein M., Vedaldi A., Bischof H., Brox T., Frahm J.-M. (2020). Square Attack: A Query-Efficient Black-Box Adversarial Attack via Random Search. Proceedings of the Computer Vision–ECCV 2020.

[37] Nicolae M.-I., Sinn M., Tran M.N., Buesser B., Rawat A., Wistuba M., Zantedeschi V., Baracaldo N., Chen B., Ludwig H. (2019). Adversarial Robustness Toolbox v1.0.0. arXiv.

[38] Sachin Kumar|Novice|Kaggle. https://www.kaggle.com/sachinkumar413.

[39] Brain Tumor MRI Dataset|Kaggle. https://www.kaggle.com/masoudnickparvar/brain-tumor-mri-dataset.

[40] SARS-CoV-2 Ct-Scan Dataset|Kaggle. https://www.kaggle.com/plameneduardo/sarscov2-ctscan-dataset.

